# Post-traumatic brain injury glioma: Characteristics, report of 2 cases report and literature review

**DOI:** 10.1097/MD.0000000000032477

**Published:** 2022-12-30

**Authors:** Kui Chen, Hugo Andrade-Barazarte, Wenjia Liang, Qingyun Zhu, Haixing Guo, Yanxin Li, Haichun Li, Rongjun Qian

**Affiliations:** a People’s Hospital of Henan University, Henan Provincial People’s Hospital, Zhengzhou, Henan Province, China; b Department of Neurosurgery, Henan Provincial People’s Hospital, People’s Hospital of Henan University, People’s Hospital of Zhengzhou University, Zhengzhou, Henan Province, China

**Keywords:** glioma, head trauma, post-traumatic glioma

## Abstract

Traumatic brain injury (TBI) as a pathogenetic factor in glioma remains controversial, and currently there is still no clear mechanism behind post-TBI glioma. Thus, we provide two case reports of post-TBI glioma contributing power to this research, and we provide a summary of the mechanisms of post-TBI glioma through literature review.

**Methods:**

We report two cases of brain gliomas that developed in the scar of a previous brain trauma. Both patients were male and both had suffered prior traumatic brain injuries (time interval 10–20 years), and postoperative pathological findings reported glioblastoma and WHO grade III glioma, respectively.

**Results:**

These two cases further support the association of between traumatic brain injury and gliomas development.

**Conclusion:**

Although the epidemiological investigation between TBI and glioma is still controversial, there are still some important aspects here that can determine the possibility between TBI and gliomagenesis. Besides, we found that the reparative response of neural stem cells and the dysregulation of inflammatory cells are timportant theories of the mechanism of post-TBI glioma.

## 1. Introduction

Glioma is the most common malignant tumor in the intracranial parenchyma, accounting for 81% of malignant intracranial tumors.^[1]^ Because of its high mortality, disability and recurrence, it brings heavy burden to families and society.^[2]^ At present, some risk factors of glioma have been identified, including chemical poisons, electromagnetic radiation, oncogenic virus infection, genetic factors, and genetic variation during development.^[3–6]^ However, due to the lack of clear pathogenesis and direct epidemiological investigation evidence, traumatic brain injury (TBI), as a risk factor for glioma, is still in a controversial state.^[7]^ Therefore, reporting more cases about post-TBI glioma and communicating the possible mechanism of post-TBI glioma are important measures to master this rare disease.

Since Manuelidis defined the diagnostic criteria for post-TBI glioma in 1978, and Moorthy proposed to join the criteria of neuroimaging in 2004, all the post-TBI gliomas need to meet the following diagnostic criteria.^[7]^ The patient is in good health before severe TBI, and there was no tumor tissue in the patient; The patient has a long incubation period, and the location of glioma should be completely corresponding to the location of TBI; There is histological evidence to prove the existence of glioma. Therefore, this study provided two cases of post-TBI glioma that meet the above criteria, and made a brief literature review, sharing our cognition and improving people’s understanding of the disease.

## 2. Case presentation

### 2.1. Case report 1

**Figure 1.**
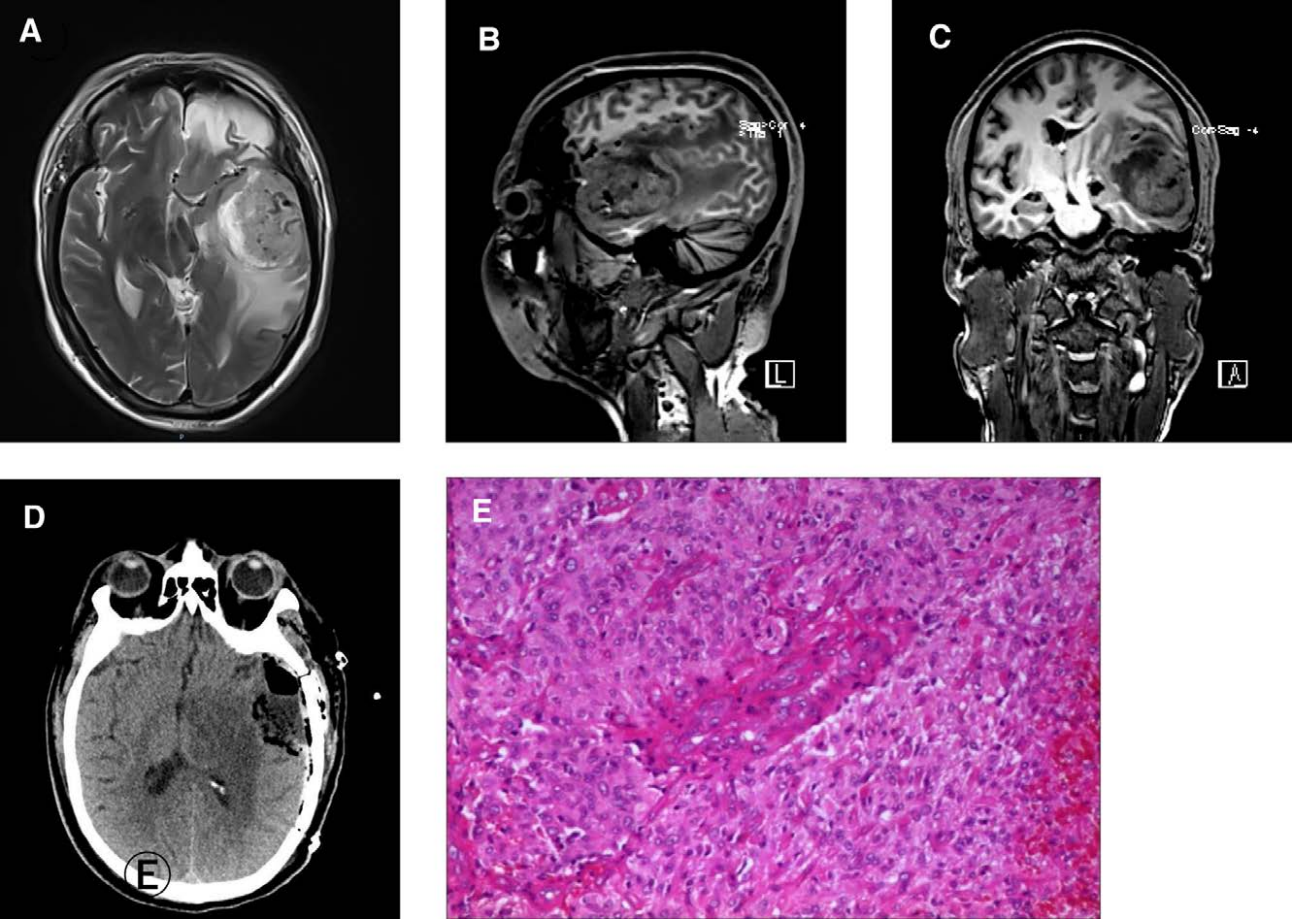
(A) Axial, (B) (sagittal), (C) (coronal) Preoperative brain MRI scan demonstrating heterogenous enhancing lesion in the left frontal and temporal lobes at the site of the previous traumatic brain injury. (D) Postoperative computed tomographic axial image demonstrating tumor resection cavity. (E) Postoperative histologic sample compatible with glioblastoma, WHO IV level.

A 57-year-old male patient was diagnosed with a severe TBI at the age of 40 years because of a severe intracranial hemorrhage and brain contusion, and was treated by neurosurgery at a local hospital. Imaging evidence and clinician diagnosis suggested the absence of neoplastic tissue at that time. The patient with worsening dizziness and persistent headache came to the Department of Neurosurgery of Henan Provincial People’s Hospital for hospitalization 17 years after the occurrence of TBI. Brain magnetic resonance imaging showed a large heterogenic enhancing lesion of approximately 5–6 cm with a necrotic center in the left temporal region, which were located at the same sites where previous TBI occurred (Fig. 1A–C). After complete preoperative evaluation and preparation, the disease variant underwent a maximal extent resection (Fig. 1D). Subsequently, histopathology of the tumor suggested that these resections were glioblastoma (Fig. 1E). After surgical resection, the patient is being treated with concurrent chemotherapy and radiotherapy following the Stupp protocol.^[8]^

### 2.2. Case report 2

**Figure 2.**
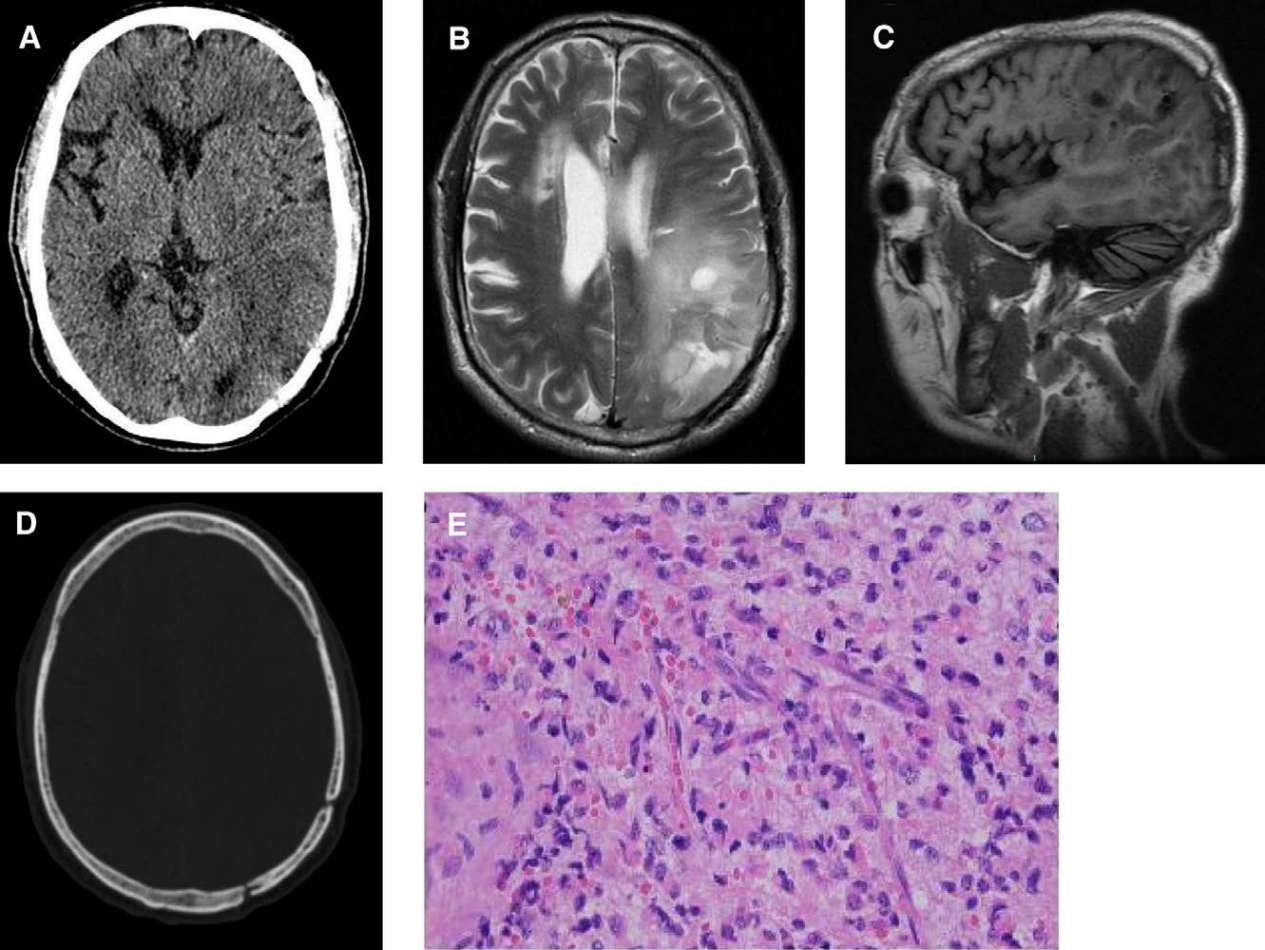
(A) Brain window, (B) (bone window) preoperative head CT showed abnormal density shadow in the left temporal occipital parietal lobe, left brain swelling, and bone discontinuity in the left occipital and parietal bones. (C) (Sagittal) and (D) (axial) preoperative MRI showing large mixed signals in the left frontotemporoparioccipital lobe, with unclear boundary, cystic degeneration, perilesional edema, and bone discontinuity in the left occipital and parietal bones. (E) Postoperative CT scan, (F) Postoperative pathological results reporting a diffuse glioma (astrocytoma) IDH mutation type, WHO III level (HE × 200), IDH = isocitrate dehydrogenase.

A 59-year-old male patient was involved in a car accident when he was 48 years old, after which he was diagnosed with severe TBI in the left parietal region. After emergency hematoma evacuation, the patient recovered well with subsequent treatment. Ten years after the injury, the patient developed seizures, requiring his admission to our department and further work-out. Computed tomographic (CT) scan showed an abnormal density shadow in the left temporo-parietal lobe, left cerebral edema, and bone discontinuity in the left occipital and parietal bones (Fig. 2A and 2B). Brain magnetic resonance imaging demonstrated a heterogeneously enhancing mass in the left parieto-occipital lobes (Fig. 2C and 2D). Areas of abnormality identified on this imaging review were consistent with sites of previous TBI. Histological analysis revealed an astrocytoma (WHO grade III) (Figure 2F). The patient had no neurological deficits and was treated with radiotherapy and chemotherapy postoperatively.

### 2.3. Literature review

To gain more insight into the epidemiology, pathogenesis, diagnostic criteria of post-TBI glioma, we performed PubMed to search from 1980 to 2021 for articles in English on post-TBI glioma by the following keyword(s): (traumatic brain injury) AND (glioma) AND (head trauma) AND (high-grade glioma) and (post-traumatic glioma). Furthermore, we scrutinized the bibliographies of the retrieved articles for additional references (Table 1).

**Table 1:** Reported cases of post-traumatic gliomas.

Authors	No.of cases	Histology	Type of trauma	Time interval (years)
Troost et al (1984)^[9]^	1	Astrocytoma grade III	Shell injury	40
Perez-Diaz et al (1985)^[10]^	2	Oligodendroglioma	Contusion	28&7
Janda et al (1987)^[11]^	1	Mixed pilocytic and fibrillary astrocytoma	Contusion	20
Di Trapani et al (1996)^[12]^	1	Mixed glioma	Head injury	*
Stendel (1997)^[13]^	1	Glioblastoma multiforme	Shell injury	48
	1	Anaplastic oligodendroglioma	After clipping aneurysm	10
Sabel et al (1999)^[14]^	1	Glioblastoma	Splinter injury	37
Henry and Rajshekhar (2000)^[15]^	1	Astrocytoma grade IV	Contusion	2
Henderson et al (2000)^[16]^	1	Anaplastic astrocytoma	Intracerebral hematoma	19
Magnavita et al (2003)^[17]^	1	Polytypic glioblastoma	Contusion	4
Moorthy and Rajshekhar (2004)^[18]^	1	Glioblastoma	Contusion	5
Salvati et al (2004)^[19]^	1	Glioblastoma	Intracerebral hematoma	7
	1	Malignant astrocytoma	Contusion	5
	1	Glioblastoma	Contusive focus	4
	1	Malignant astrocytoma	Intracerebral hematoma	7
Anselmi et al (2006)^[20]^	1	Glioblastoma multiforme	Intrathecal hematoma	20
	1	Glioblastoma multiforme	Contusion	15
Hebert-Blouin (2010)^[21]^	1	Spinal cord anaplastic astrocytoma	Brachial plexus injury	5
Zhou (2010)^[22]^	1	Glioblastoma multiforme	Intracerebral hematoma	10
Han Z et al (2013)^[23]^	1	Glioblastoma multiforme	Intracerebral hematoma	9
Tyagi V et al (2016)^[24]^	2	Glioblastoma	Contusion	11&7
Simińska, D et al(2018)^[25]^	1	Glioblastoma	Contusion	2
Juškys, R et al(2020)^[26]^	1	Glioblastoma	Contusion	4
Present	1	Glioblastoma multiforme	Contusion	17
	1	Astrocytoma grade III	Contusion	10

* not given, data from abstract.

## 3. Discussion

There is currently debate as to whether TBI is a risk factor in glioma pathogenesis, which is also in the state of 2-level differentiation among neurosurgical experts. Some views showed that there is no obvious causal relationship between TBI and gliomagenesis. For example, the results of a large study in Denmark demonstrated a significantly higher probability of gliomagenesis within the first year of head injury, probably because of the presence of glioma before injury. However, there was no risk here that cerebral injury was able to increase gliomagenesis after excluding the first year of follow-up.^[27]^ Similar studies have failed to identify TBI as a risk factor for gliomagenesis.^[28,29]^ However, there are still many scientists who hold the opposite view and believe that there is a clear causal relationship between TBI and gliomagenesis. Several case reports and case-control studies have reported an increased risk for primary malignant brain tumors after head injury.^[19,23,25,30–32]^ For example, a cohort study including 5007 patients found a higher incidence rate of malignant brain tumors (6.29 per 10000 persons-years) in patients who had suffered a TBI as compared to the control groups (1.25 per 10000 person‐years). Additionally, these authors reported a statistically significant association between TBI severity and brain tumor development.^[33]^ Besides, Preston Martin et al, and Monteiro et al, emphasized that there is a positive relationship between the severity and frequency of head injury and subsequent tumor formation.^[34,35]^ It is precisely the current controversial views that TBI is a risk factor for glioma, and more studies about the relationship between TBI and glioma are proposed to be more meaningful. Therefore, this article provided 2 case reports of glioma formation following TBI, contributing a strength to medical research.

Currently, after continuous refinement over the course of several decades of post-TBI glioma treatment by generation after generation of neurosurgeons, diagnostic criteria for post-TBI glioma are proposed. Zulch and Manuelidis reported specific diagnostic criteria to determine a direct causal relationship between head trauma and glioma formation.^[36,37]^ Firstly, Zulch established the following criteria in 1974: Patient in good health before suffering the TBI; Head trauma severe enough to cause a contusion or a secondary reparative process; Location of the trauma and tumor should correspond exactly one to another; Time interval between trauma and tumor appearance of at least 1 year, a longer period increased the likelihood of a causal relationship; Histological proof of tumor; Trauma of external force.^[38]^ Then, Manuelidis added the following diagnostic criteria: Histological proof of traumatized brain; Clear differentiation between bleeding, scar and secondary edema caused by the tumor than caused by trauma; Tumor tissue in direct continuity with the traumatic scar, not merely in its vicinity or separated by a narrow zone of healthy or slightly anomalous tissue. Subsequently, reports of post-TBI gliomas were required to meet all of the above diagnostic criteria. However, histological biopsy was not performed in the surgical treatment of TBI at that time, which might be an important reason hindering the development of post-TBI gliomas research. Fortunately, with the popularity of imaging technology, neurosurgeons could not only judge whether patients had tumor tissue present before the occurrence of TBI, but also made clear that gliomas were formed in the place of brain injury.^[15,22–24]^ Therefore, this study also provided two male patients with post-TBI gliomas following the above diagnostic criteria. In this study, we found that both patients had severe TBI injuries with contusions and intracerebral hemorrhage before glioma formation, and both existed for an interval period of more than 10 years. In short, based on the above literature review and our findings, we preferred to consider TBI as an pathogenetic factor in gliomagenesis.

Although there is no direct experimental evidence to prove the pathogenesis of post-TBI glioma, there are still several potential theories that the possible mechanisms of post-TBI glioma was proposed. One of these emphasized that the inflammatory response after TBI is associated with the oncogenic transformation of progenitor cells and neural stem cells, which migrate to the site of injury as a repair mechanism.^[24]^ This theory is based on evidence of tumor development in other organs following strong inflammatory response, such as pancreatitis or hepatitis.^[39]^ Besides, neural stem cells and descendent progenitor cells chemotactically migrate to the injury side and actively participate in the repair and recovery of the lesion.^[40,41]^ Therefore, gliomas may occur from the transformation of these cells. The other theory tended to favor the idea that TBI caused the disruption of the brain microenvironment, which gave rise to the disturbed expression of immune cells in brain to create a tumor promoting environment. During the acute phase of severe TBI, there is continuous mobilization of resident brain microglia and myeloid inflammatory cells to the injury site. Additionally, due to the disruption of the blood brain barrier during the trauma, peripheral neutrophils, monocytes and eosinophils are recruited to the site within hours and can remain there during extended periods of time.^[25]^ Here, these cells can contribute to oncogenesis either by secreting growth factors and cytokines that have mitogenic effects on neural stem and progenitors cells or by generating reactive oxygen species which have mutagenic properties.^[24,25,42,43]^ Regretfully, as there are currently no well constructed in vitro and in vivo models of post-TBI glioma, none of the theories has direct evidence to demonstrate. Therefore, neurosurgeons also need more efforts to figure out the mechanisms behind the post-TBI glioma.

## 4. Conclusions

The causal relationship between head injury and glioma remains controversial. Therefore, to determine direct connection between TBI and glioma, physicians should carefully inquire and fully understand patient’s previous history of head trauma. For patients with a history of TBI, we recommend regular follow-up visits to monitor symptoms such as headaches, nausea, vomiting, epilepsy, and vision loss. Early actions and radiological studies on symptomatic patients could improve the early detection rate of glioma. We hope that the well-established follow-up system will help us to find a clear association between TBI and glioma in our following studies. Besides, with the help of literature review, we identified that post-TBI glioma may occur through the reparative response of neural stem cells and dysregulation of inflammatory cells.

## Acknowledgments

The authors would like to express their gratitude to Medical Image Center, Henan Provincial People’s Hospital, for their helpful information regarding radiological image acquisition.

## Author contributions

KC and Hugo wrote the manuscript. RQ, YL, HL, and HG performed the surgery. WL and QZ drafted the manuscript. All authors read and approved the final manuscript.

**Conceptualization:** Yanxin Li, Rongjun Qian.

**Data curation:** Kui Chen.

**Formal analysis:** Yanxin Li.

**Investigation:** Wenjia Liang.

**Methodology:** Hugo Andrade-Barazarte, Wenjia Liang.

**Software:** Qingyun Zhu.

**Supervision:** Hanchun Li.

**Validation:** Qingyun Zhu, Haixing Guo, Hanchun Li.

**Writing—original draft:** Kui Chen.

**Writing—review and editing:** Hugo Andrade-Barazarte, Rongjun Qian.
